# Assessment of gastric pouch blood supply with indocyanine green fluorescence in conversional and revisional bariatric surgery: a prospective comparative study

**DOI:** 10.1038/s41598-023-36442-4

**Published:** 2023-06-06

**Authors:** Francesco Mongelli, Fabio Garofalo, Pietro Giacopelli, Martino Munini, Francesco Volontè, Michele Marengo

**Affiliations:** 1 Department of Surgery, Bellinzona e Valli Regional Hospital, EOC, Via Gallino 12, 6500 Bellinzona, Switzerland; 2 Faculty of Biomedical Sciences, Università Della Svizzera Italiana, 6500 Lugano, Switzerland; 3 Department of Surgery, Lugano Regional Hospital, EOC, 6900 Lugano, Switzerland; 4 Department of Surgery, Mendrisio Regional Hospital, EOC, 6850 Mendrisio, Switzerland; 5 Department of Surgery, Sant’Anna Clinic, 6900 Lugano, Switzerland; 6 Department of Surgery, Locarno Regional Hospital, EOC, 6600 Locarno, Switzerland

**Keywords:** Obesity, Gastrointestinal diseases, Metabolic disorders

## Abstract

Our study aimed to evaluate the usefulness of indocyanine green (ICG) angiography during conversional or revisional bariatric surgery. We prospectively enrolled all patients scheduled for reoperative bariatric surgery with gastric pouch resizing and ICG assessment and we compared them with a retrospective series of similar patients who did not receive ICG. The primary outcome was the rate of intraoperative change in the surgical strategy due to the ICG test. We included 32 prospective patients receiving intraoperatively an ICG perfusion test and 48 propensity score-matched controls. The mean age was 50.7 ± 9.7 years, 67 (83.7%) patients were female, and the mean BMI was 36.8 ± 5.3 kg/m^2^. The patient characteristics were similar in both groups. The ICG angiography was successfully conducted in all patients, and no change of the surgical strategy was necessary. Postoperative complications were similar in both groups (6.2% vs. 8.3%, *p* = 0.846), as well as operative time (125 ± 43 vs. 133 ± 47 min, *p* = 0.454) and length of hospital stay (2.8 ± 1.0 vs. 3.3 ± 2.2 days, *p* = 0.213). Our study suggested that ICG fluorescence angiography might not have been useful for assessing the blood supply of the gastric pouch in patients who underwent reoperative bariatric surgery. Therefore, it remains uncertain whether the application of this technique is indicated.

Bariatric surgery has been spreading over the last few years, as severe obesity is constantly increasing^1,2^. This resulted in a growing need to perform reoperative bariatric surgery for insufficient weight loss, weight regain, severe gastroesophageal reflux, or solid food intolerance after primary bariatric surgery^3^. However, reoperative surgery, either revisional or conversional, is associated with an increased risk of postoperative complications compared to primary bariatric surgery^4,5^. More specifically, the risk of leakage ranges from 0.8 to 6 percent for primary bariatric surgery^6,7^, while for reoperative surgery it approaches 35 percent^5^ and the most frequent site is the gastro-jejunal anastomosis. Anastomotic leakage is still the most common life-threatening complication and is associated with increased hospital stays and costs^5,8,9^.

Fluorescence can be detected thanks to special cameras that are sensitive to the near-infrared spectrum. The indocyanine green (ICG) absorbs near-infrared light at 800 to 810 nm wavelengths. This fluorophore emits fluorescence at 830 nm when bound to tissue proteins if excited and allows the determination of blood supply^10,11^. To date, ICG fluorescent angiography has been demonstrated to be useful in colorectal surgery to assess tissue blood perfusion^12^. A relevant percentage of anastomotic leakage is thought to be caused by insufficient blood supply, which can be evaluated by ICG angiography^13^. Recent works in colorectal surgery have postulated that the use of indocyanine green may lead to a change in choosing the optimal site for anastomosis, thus reducing the rate of anastomotic leakage^14^. However, the use of indocyanine green in bariatric surgery has never been considered mandatory or necessary like in colorectal surgery, as the blood supply of the stomach is generally adequate^10,11,15^. Few studies have assessed the usefulness of ICG fluorescence in primary or reoperative bariatric surgery^11,16–18^.

We hypothesized that the use of ICG angiography during reoperative bariatric surgery, either conversional or revisional, could lead to a change in the intraoperative surgical strategy and could be best applied to cases where a gastric pouch resizing was necessary. Our study aimed to evaluate the usefulness of intraoperative ICG assessment of the anastomotic vascular perfusion during reoperative bariatric surgery.

## Materials and methods

At our institution, a tertiary, and referral center for bariatric surgery, we prospectively enrolled from April 2021 to October 2022 all patients scheduled for reoperative bariatric surgery (i.e., conversional and revisional) to undergo surgery with an ICG assessment of the gastric pouch. Also, we retrieved from a prospectively maintained database for bariatric surgery a series of patients who underwent already reoperative bariatric surgery without the ICG test. We included only patients undergoing reoperative bariatric surgery with gastric pouch resizing and gastro-jejunal anastomosis, namely cases where the gastric blood supply could have been altered. Patients were excluded in case of known allergy to ICG or when the gastric pouch resizing was not deemed necessary.

This study was approved by the local ethic committee (Comitato etico cantonale Ticino, 2021-00722 CE 3853). Written informed consent and consent for publication were obtained before inclusion. All methods were performed in accordance with the relevant guidelines and regulations. Strengthening the reporting of observational studies in epidemiology (STROBE) guidelines were followed.

Data collected were age, gender, body mass index (BMI, expressed in kg/m^2^), the indication to surgery (i.e., insufficient weight loss, weight regain, reflux), type of initial bariatric operation, operative time, type of surgery, need to change the surgical strategy according to the ICG test, adverse event due to ICG administration, intraoperative complications, postoperative complications graded with the Clavien-Dindo grading system^19^ and length of hospital stay.

The primary outcome was the rate of intraoperative change of the surgical strategy due to the ICG test. Secondary outcomes were the rate of postoperative anastomotic leakage and complications, operative time, and the length of hospital stay.

ICG fluorescent angiography to test the intraoperative blood supply was introduced in our institution in 2014 for colorectal surgery and since 2021 has been applied also to reoperative bariatric surgery. The ICG protocol was the following: 0.1 mg of ICG (VERDYE®) / (kg of body weight) was administered intravenously and the laparoscopic camera was then switched to near-infrared vision. The blood supply of the resized gastric pouch was assessed to check the blood supply on all gastric pouch surfaces and was rated as adequate or inadequate.

Insufficient weight loss was defined as an excess weight loss percentage (EWL%) inferior to 50% 18 months after surgery^20^. Weight regain was defined as a significant regain of weight after an initially successful weight loss^20^. We defined gastric pouch resizing as any volume reduction carried out with linear staplers applied to either the gastric tube after sleeve gastrectomy (SG) or the gastric pouch after gastric bypass (GB)^21^. Conversional surgery was defined as the conversion of a previous bariatric operation into another one (e.g., SG to GB, gastric banding to GB), while revisional surgery was defined as a redo surgery where the initial configuration was maintained (e.g., gastric pouch resizing and distalization for GB)^5,22^. Finally, the need to intraoperatively change the surgical strategy was defined as any change of the planned surgical steps that occurred after and were due to the ICG fluorescence test on the gastric pouch.

All patients scheduled for surgery were multidisciplinary assessed by surgeons, physician nutrition specialists, dieticians, anesthesiologists, and others according to comorbidities. Patients were operated on in a supine position with open legs, pneumatic stocking, antibiotic prophylaxis, and a laparoscopic approach was used in all cases. Postoperatively, patients received basic analgesia, rescue medications, and respiratory physiotherapy. All patients could start drinking on the day of surgery and receive semi-solid food on the first postoperative day. Patients were discharged once having achieved good mobilization, feeding, and pain control. After discharge, patients were followed-up after one and four weeks.

### Statistical analysis

Descriptive statistics were presented as absolute frequencies for categorical variables and mean with standard deviation (SD) for continuous variables. The comparison of categorical variables between the two groups was carried out with the chi-squared test, while for continuous variables the Student t-test was used. For comparisons of interest also standardized mean difference (SMD) and odds ratio (OR) with 95% confidence interval (95%CI) were provided. As data regarding the need for intraoperative surgical strategy change due to ICG tests in reoperative bariatric surgery lacks, we assumed a 9% rate of surgical strategy change. To achieve a 95% probability to detect at least one event 31 patients were considered necessary. Unfortunately, also data regarding postoperative complications in patients receiving or not the ICG test lack, therefore, an intervention/control group sample size was not estimable. A propensity score matched (PSM) analysis was conducted with a 1:1.5 ratio to minimize the effect of confounders according to age, gender, BMI, comorbidity, and type of surgery. MedCalc® Statistical Software version 19.6 was used (MedCalc Software Ltd, Ostend, Belgium; https://www.medcalc.org; 2020).

## Results

**Figure 1:**
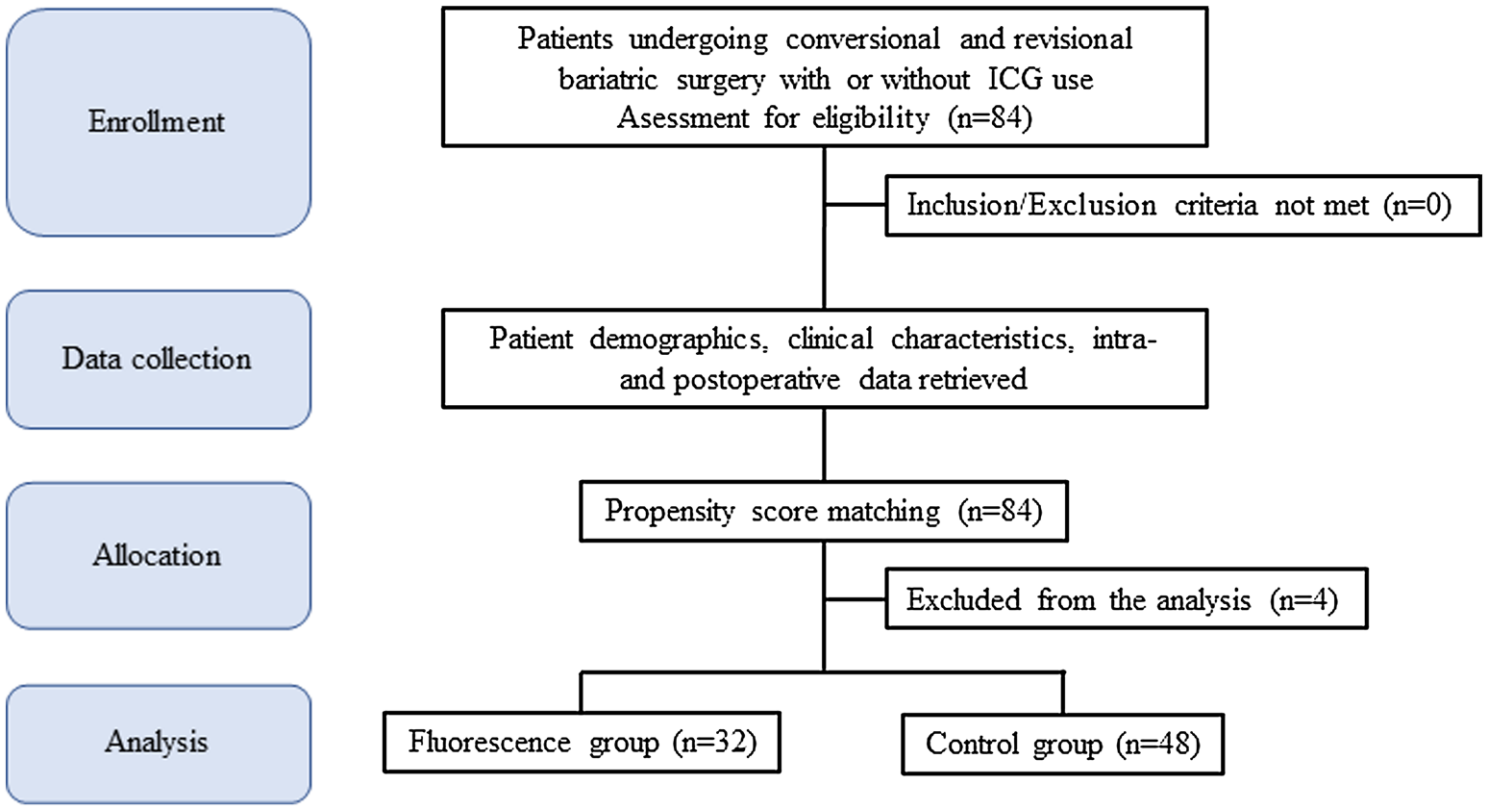
Study flow-chart.

During the study period, we screened 84 consecutive patients who underwent reoperative bariatric surgery with gastric pouch resizing. Of them, 32 were prospectively enrolled and scheduled for surgery with an intraoperative ICG assessment of the gastric pouch perfusion. For the control group, we screened a prospective database of patients undergoing bariatric surgery at our institution, and we identified 54 patients who underwent reoperative bariatric surgery with gastric pouch resizing. Subsequently, we matched patients of the two groups with a propensity score that excluded 6 patients of the control group. Therefore, the final analysis was carried out on 80 patients, 32 receiving and 48 not receiving the ICG test intraoperatively (Fig. 1). The overall mean age was 50.7 ± 9.7 years, 67 (83.7%) patients were female, and the mean BMI was 36.8 ± 5.3 kg/m^2^. Details of demographics and comorbidities are reported in Table 1.

**Table 1:** Patients’ characteristics.

Variables	Fluorescence group	PSM control group	*p*
	N = 32	N = 32	
Age, years (SD)	50.8 (9.5)	50.7 (10.0)	0.962
Gender, female (%)	26 (81.2)	41 (85.4)	0.623
BMI, kg/m^2^ (SD)	37.4 (6.4)	36.3 (4.4)	0.382
Active smoking, n (%)	9 (28.1)	14 (29.2)	0.920
Comorbidities			
Hypertension, n (%)	6 (18.8)	5 (10.4)	0.292
OSAS, n (%)	10 (31.2)	11 (22.9)	0.409
Dyslipidemia, n (%)	2 (6.2)	3 (6.2)	1.000
Diabetes mellitus, n (%)	6 (18.8)	4 (8.3)	0.170
Arthralgia, n (%)	4 (12.5)	2 (4.2)	0.168
Depressive syndrome, n (%)	5 (15.6)	7 (14.6)	0.899
Reflux, n (%)	11 (34.4)	11 (22.9)	0.264
Indication to surgery			
Insufficient weight loss, n (%)	5 (15.6)	6 (12.5)	
Weight regain, n (%)	6 (18.8)	11 (22.9)	0.862
Reflux, n (%)	21 (65.6)	31 (64.6)	

*OSAS* obstructive sleep apnea syndrome.

Dichotomous variables are expressed as a number with a percentage. Continuous variables are expressed as mean with standard deviation (SD).

In the fluorescence and control groups, indications to surgery were insufficient weight loss in 5 (15.6%) vs. 6 (12.5%) patients, weight regain in 21 (65.6%) vs. 31 (64.6%), and reflux in 6 (18.8%) vs. 11 (22.9%). In the fluorescence group 19 (59.4%) cases were of conversional surgery, from gastric banding in 5 (15.6%) patients, and from SG in 14 (43.7%) patients. In the control group 32 (66.7%) cases were of conversional surgery, from gastric banding in 14 (29.9%) patients, and from SG in 18 (37.5%) patients. The other 13 (40.6%) vs. 16 (33.3%) cases were all of the revisional surgery and were all cases of gastric pouch resizing and distalization after GB. The types of reoperative surgery were equally distributed in both groups (*p* = 0.395).

**Figure 2:**
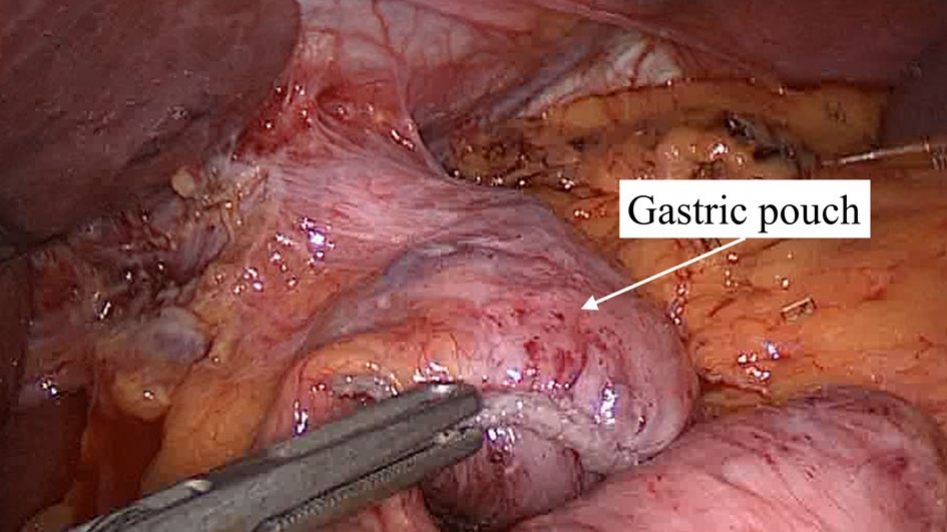
Intraoperative image showing a resized gastric pouch.

**Figure 3:**
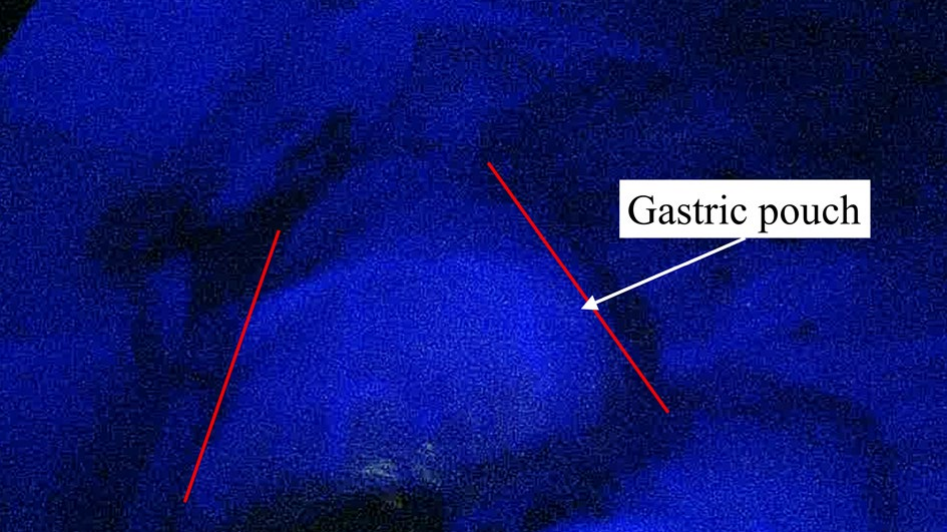
Intraoperative image showing the good blood supply of the gastric pouch with indocyanine green fluoroscopy. The red lines delimitate the gastric pouch.

During the surgery, the ICG angiography was successfully carried out in all 32 prospective patients with no adverse event. In all cases, the blood supply of the gastric pouch was judged adequate, without any ischemia area. In no patient, there was the need to change the intraoperative strategy according to the ICG test performed. Figures 2 and 3. No intraoperative complications occurred in both groups. Operative time was 125 ± 43 min in the fluorescence group vs. 133 ± 47 min in the control group (SMD 0.17, 95%CI −0.28 to 0.63, *p* = 0.454).

Within 30 days after surgery, two (6.2%) vs. 4 (8.3%) complications occurred (OR 1.36, 95%CI 0.23–7.92, *p* = 0.730). In the fluorescence group, we recorded one case of stenosis of the gastro-jejunal anastomosis that was successfully treated with repeated endoscopies and balloon dilatation (graded 3 according to the Clavien-Dindo classification). One case of leakage of the gastro-jejunal anastomosis occurred and required reoperation and a prolonged stay in the intensive care unit (grade IV Clavien-Dindo). In both cases, the intraoperative ICG fluorescent angiography demonstrated an adequate blood supply of the whole gastric pouch. In the control group, we recorded one case of severe gastrojejunal anastomosis bleeding that required reoperation and intensive care unit admission (grade IV Clavien-Dindo). Another patient had an anastomotic leakage that required endoscopic treatment (grade III Clavien-Dindo). One patient developed an anastomotic stenosis that required endoscopic dilatations (grade III Clavien-Dindo). One patient had a mild pancreatitis (grade I Clavien-Dindo) Table 2.

**Table 2:** Intra- and postoperative results.

Variables	Fluorescence group N = 32	PSM control group N = 48	*p*
Need of surgical strategy change, n (%)	0	–	–
Intraoperative complications, n (%)	0	0	–
Operative time, min (SD)	125 (43)	133 (47)	0.454
Postoperative complications	1 CD grade 3 1 CD grade 4	1 CD grade 1 2 CD grade 3 1 CD grade 4	0.846
Length of hospital stay, days (IQR)	2.8 (1.0)	3.3 (2.2)	0.213

Dichotomous variables are expressed as numbers with percentages. Continuous variables are expressed as mean with standard deviation (SD)

*CD* Clavien-Dindo.

Finally, the length of hospital stay was 2.8 ± 1.0 days in the fluorescence group and 3.3 ± 2.2 days in the control group (SMD 0.29, 95%CI −0.17 to 0.74, *p* = 0.213).

## Discussion

In our study on 32 patients prospectively undergoing reoperative bariatric surgery with ICG perfusion assessment of the gastric pouch, no change of the surgical strategy was deemed necessary. These patients, compared with a retrospective control group of 48 patients with similar characteristics and selected with a PSM analysis had similar postoperative courses.

Reoperative bariatric surgery, either revisional or conversional, is associated with a higher rate of postoperative complications compared to primary surgery^5,23^. Such complications are eventually due to intrabdominal adhesions, altered tissue quality and anatomy, the presence of foreign materials and disposals, and comorbidities^4^. An adequate blood supply of the anastomoses is crucial for uneventful tissue healing and might be altered in the case of previous operations. The gastro-jejunal anastomoses are particularly at risk of leakage in revisional and conversional bariatric surgery for several reasons, being an adequate blood supply one of them. Many articles on colorectal surgery have demonstrated that the blood supply assessment cannot be based on the bowel color change alone and ischemic areas are likely to be evident after a few hours^24^. The same concept can be applied in reoperative bariatric surgery, where altered tissue perfusion might be an issue.

Severe side effects due to the administration of ICG are mostly related to allergic reactions and are extremely rare^16,18^. In our series, no ICG-related complications were recorded, and also the intraoperative time dedicated to the test was low (a few minutes). The safety and feasibility demonstrated in our study were also reported by many articles in the literature^15–18,25,26^.

In our study, we did not find any case where ICG use led to a change in the surgical strategy. Indeed, patients who developed complications, namely gastrojejunal stenosis or leak, occurred both in patients with a normal ICG perfusion test. Both complications can be eventually caused by an inadequate blood supply, however, this was not the case for our patients. We also compared our prospective series of patients to a retrospective control group with similar characteristics and no difference in postoperative complications was noted. Recently, Balla et al.^16^ published a series of 13 patients who underwent either primary or conversional bariatric surgery. The authors scored the ICG-tested vascular supply and found that in two out of four conversional cases (from SG to GB) a change of the surgical strategy was deemed necessary. Garofalo et al.^15^ published a video report assessing the safety and feasibility of ICG use during a two-step conversion from GB to single anastomosis duodeno–ileal bypass with sleeve gastrectomy. The authors found that, although ICG was useful to assess the blood supply, no change in the surgical strategy was necessary. Similar conclusions were also reported by Olmi et al.^25^ in 2019 in a patient who underwent an SG combined with Rossetti fundoplication. Other studies published in the literature assessed the usefulness of ICG during primary bariatric surgery, mainly during SG. Di Furia et al.^26^ published a series of 45 patients undergoing SG and tested intraoperatively with ICG. The authors found that an adequate blood supply was demonstrated in all cases and the etiology of leakage is likely to be multifactorial. Similarly, Spota et al.^18^ published a study including 129 bariatric operations from the European-Fluorescence Imaging-Guided Surgery (EURO-FIGS). The authors found that the use of ICG, although beneficial for the surgeons’ sense of confidence, was unrelated to the anastomosis site.

Our study has many limitations. The most relevant one is the small sample size. However, according to our estimation, with a sample size of 31 patients, we had a probability > 95% of finding at least one case where the intraoperative strategy would have changed. Also, no data regarding postoperative complications in patients receiving or not the ICG test are available in the literature, therefore, no sample size calculation could be carried out. Due to the small sample size and the study design, no thorough statistical analysis was carried out and no correlation between the ICG perfusion test and postoperative complication could be assessed. The lack of randomization is another issue, however, we tried to compensate for with a PSM analysis. Our results should be taken as descriptive and be eventually helpful for designing larger multicentric studies. Despite its limitations, our study is the first one assessing if the intraoperative ICG perfusion test would be helpful in patients undergoing reoperative bariatric surgery with gastric pouch resizing.

In conclusion, considering the remarkably low level of evidence and limitations, our study suggested that ICG fluorescence angiography might not have been useful for assessing the gastric pouch blood supply in patients who underwent reoperative bariatric surgery. As a result, it is uncertain whether the application of this technique is indicated. Additionally, it was important to recognize that other factors, rather than solely the gastric pouch blood supply, could have potentially played a more substantial role in the development of postoperative complications.

## Data Availability

The dataset analysed during the current study is available from the corresponding author on request.
